# Cost-Effectiveness Analysis of Treatment for Metastatic Renal Carcinoma in Romania

**DOI:** 10.25122/jml-2018-0069

**Published:** 2018

**Authors:** Alin Liviu Preda, Dana Galieta Mincă

**Affiliations:** 1. Public Health and Management Department, Carol Davila University of Medicine and Pharmacy, Bucharest

**Keywords:** cost-effectiveness, cost-utility, renal cancer, reimbursement

## Abstract

**Rationale:** In recent years, the cost of several treatment options for renal cancer have been supported by the Romanian healthcare system for both first- and second-line therapies. First-line alternatives through real-life efficacy and amplitude of adverse reactions may influence the efficacy and costs of patients treated with second-line treatment.

**Objective:** Estimation of the cost-effectiveness and cost-benefit ratio for first-line treatment alternatives: Sunitinib and Pazopanib from the payer’s perspective in the Romanian healthcare system.

**Methods and Results:** We developed a Markov model to calculate the cost-effectiveness and cost-benefit ratio for 2 cohorts of patients using the results from the COMPARZ study for efficacy (progression-free survival, general survivability and quality of life) and safety and costs from national hospital databases. For an estimated population of 800 patients, Pazopanib has a quantified benefit of 7.19 years in progression-free survival, 11.71 life years gained and 8.97 years of quality-adjusted life-years compared to Sunitinib. The analysis is limited by the accuracy of the national data used and the transposition of general data on efficacy and safety at the local level.

## Introduction

### Epidemiology

As it is one of the most studied types of cancer, many publications regarding renal carcinoma (RC) refer to epidemiology, morphology, therapeutic options and the economic and social burden. Worldwide, the annual incidence of RC increased from 1.5 to 5.9 cases per 100.000 inhabitants [1]. Statistical models indicate that the incidence increased by 1.1% over the past 10 years while the mortality rate for this cause decreased by 0.7% between 2004 and 2015 [2]. The highest incidence is found in Europe and North America. In 2012, 84.400 cases were estimated in the European Union with 34.700 corresponding deaths [3]. Mortality rates are stable in most European countries [4], and in Romania, the prevalence of RC over the last 5 years is around 5.400 cases with an annual incidence of 2000 cases [5].

### Etiology

The precise etiology of RC cannot be determined accurately, but several assumptions have been made regarding environmental, physiological and genetic factors. The most frequent factors quoted in the literature are smoking and obesity. Smoking is associated with a 2-fold higher risk increase to develop kidney cancer, this risk being directly proportional to the number of cigarettes smoked [6]. Obesity is closely linked to the occurrence of RC, especially among women [7]. Hypertension and frequent use of analgesics are other risk factors for RC [8].

The costs of treatment for patients diagnosed with RC in Romania continue to increase mainly due to the latest reimbursed therapeutic alternatives. As the financial burden continues to grow, it is essential to understand the therapeutic value of the different treatment alternatives available. This study aims to quantify the cost-effectiveness and cost-utility ratio for the two existing therapeutic alternatives (Sunitinib and Pazopanib) for treating RC as the first-line option from the payer’s perspective.

## Materials and Methods

We developed a Markov model to calculate the cost-effectiveness and cost-utility ratios for two cohorts of patients initiated in the first line with either Sunitinib or Pazopanib. In order to compare the two alternatives, we have used indirect clinical efficacy analyses for the two alternatives. The results were quantified in Progression-Free Survival (PFS), Life-Years Gained, and Quality-Adjusted Life-Years (QALY) for Cost-Benefit Analysis. The results were expressed in incremental cost-effectiveness ratios (ICERs) and incremental cost-utility ratios (ICURs).

### Structure of model, population and comparator

**Figure 1:**
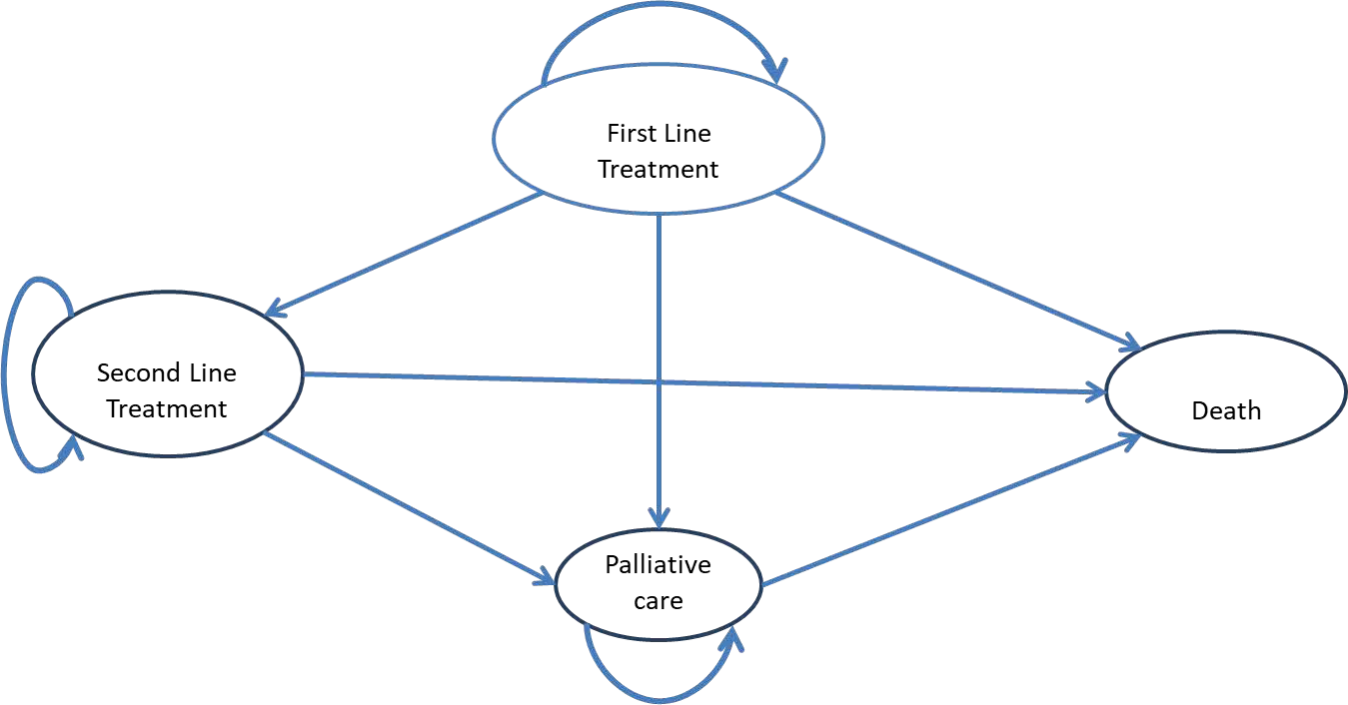
Model structure

We used a cohort model that transitions from one state to another and that simulates the natural progression of the disease. For each pre-defined status in the model, we calculated health costs and outcomes for a cohort of 800 patients over one year. We chose the group based on existing data on the number of metastatic cancers treated and reported by the National Health Insurance House as well as international data extrapolated locally. The mathematical model has been developed in Microsoft Excel and follows the logical structure in Figure 1. Characteristics of the cohort of patients are identical to those of the patient population included in the COMPARZ study [9]. The discount level for both costs and utilities was estimated at 3%. The two drugs used for the study are as follows:

– Sunitinib is a drug used in oncology that has a small molecular structure. It acts by inhibiting the enzyme activity of the tyrosine kinase receptor that is over-expressed in metastatic RC. This drug has been marketed in Romania by Pfizer Romania since 2008.
– Pazopanib has the same mechanism of action (tyrosine kinase inhibitor) with an effect on angiogenesis. This drug has been marketed in Romania by Novartis since 2016.

### Efficacy

As defined in the COMPARZ clinical trial, the efficacy included in the model is defined as follows:

– Progression-free survival is defined as the time interval between the time of randomization and the time of tumor progression documentation or death as a result of any cause.
– Overall survival is defined as the time interval between the time of randomization and the date of death of any cause.

For patients considered for second-line of treatment, the following options were analysed out of the medications reimbursed in Romania in 2017: Sorafenib, Everolimus, Bevacizumab, Temsirolimus, and Axitinib.

**Table 1:** PFS and OS as resulted from COMPARZ trial

	PFS	OS
**Pazopanib**	8.3 months (Confidence interval 95% [CI]: 8,3-10,9)	28.4 months (95% [CI]: 26,2-35,6)
**Sunitinib**	9.5 months (Confidence interval 95% [CI]: 8,3-11,1)	29.3 months (95% [CI]: 25,3 -32,5)

### Adverse Effects and Utilities

The adverse effects and the value of the disutility corresponding to each type of adverse effect were used in accordance with the published studies: [9–11] and are summarized in Table 2.

In the COMPARZ study, data on quality of life was recorded using the EQ-5D questionnaire. The questionnaire was administered on day 1, day 28 of each treatment cycle, and completion of the study for each patient. The baseline on day 1 of the study was 0.76 in both the test group and the control group. Progression under treatment decreases the value of utilities by 0.1252, but the administration of the second-line treatment brings a positive impact of 0.005 [12]. Progression of disease in the second-line of treatment decreases utility by 0.075 and is equivalent to the utility for palliative care.

### Costs

The treatment costs were calculated in accordance with the therapeutic guidelines regulated by the Ministry of Health Order (1301/2008) and the National Medicines Catalogue of April 2018 as can be seen in Table 3.

The costs for the use of health services and the treatment of adverse events were calculated taking into account the available data from the DRG database and the framework contract tariffs for contracting health services as follows (Table 4):

## Results

For a cohort of 800 patients analysed over a 1-year period at a discount rate of 3%, according to PFS and OS data from the COMPARZ study and according to the cost data from the DRG database and standard tariffs for medical services, Pazopanib has a quantified benefit in years of progression-free survival of 7.19; a total of 11.71 years gained and 8.97 quality-adjusted life-years gained compared to Sunitinib as can be seen in Table 5.

**Figure 2:**
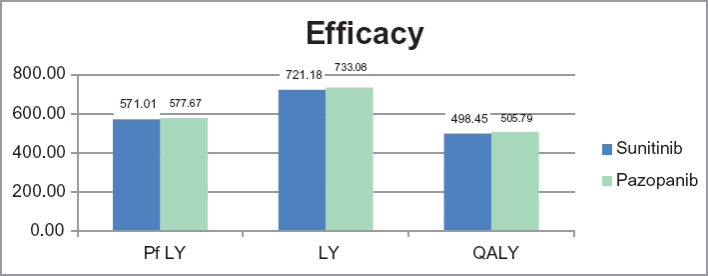
Efficacy of Sunitinib vs. Pazopanib

From the efficacy point of view, Pazopanib is dominant over Sunitinib in the patient population in which the analysis was performed due to differences in safety (Figure 2).

**Table 2:** Summarizing the frequency and duration of the adverse effects used in the model and the disutility associated with each adverse effect

Grade 1 & 2 adverse effects	Sunitinib	Pazopanib	Disutility caused by adverse effects	Average duration of the event (days)
**Total number of therapy cycles administered**	3288	3324		
**Number of patients enrolled in the study**	548	554		
**Total number of recorded events**	6837	6028		
**Fatigue/asthenia**	925	875	−0.0007	72.04
**Stomatitis**	344	177	−0.0018	33.60
**Hypertension**	246	303	–	56.92
**Thrombocytopenia**	638	434	−0.0105	23.52
**Neutropenia**	608	414	0.0223	57.35
**Nausea/Vomiting**	1101	1134	−0.0151	30.90
**Diarrhea**	1345	1473	−0.0261	64.15
**Anemia**	505	281	−0.0114	29.63
**Mouth-to-mouth syndrome**	676	420	0.0018	68.63
**Proteinuria**	80	113	−0.018	55.40
**Rash**	300	230	–	–
**Anorexia**	71	175	−0.0082	49.85
**Grade 3 & 4 adverse events**				
**Total number of observed events**	626	482		
**Fatigue/asthenia**	140	88	−0.1237	18.29
**Stomatitis**	8	10	−0.0018	33.60
**Hypertension**	89	108	−0.0084	36.06
**Thrombocytopenia**	117	28	−0.024	14.02
**Neutropenia**	109	58	0.0223	57.35
**Nausea/Vomiting**	11	16	−0.0532	11.07
**Diarrhea**	44	73	−0.0261	64.15
**Anemia**	40	21	–	12.85
**Mouth-to-mouth syndrome**	63	58	0.0141	20.00
**Proteinuria**	0	0	−0.018	55.40
**Rash**	4	10	–	–
**Anorexia**	1	13	−0.0082	49.85
**Source**	COMPARZ	COMPARZ		

Regarding the progression-free survival, the difference was 7.19 years for the 800 patients considered for the two scenarios, in favor of Pazopanib. The differences are in favor of Pazopanib also for Life-Years Gained and for Quality-Adjusted Life-Years. The cost difference between the two alternatives is 6.5 million RON over one year. The incremental cost for a QALY is 722.3 RON in favor of Pazopanib, as shown in Table 4.

### Sensitivity analysis

The sensitivity analysis for the two alternatives was done by adjusting between -20% and + 20% for 138 variables that can influence the outcome. The largest variations are resulting from:

– Pazopanib dose variation (200 vs. 400 mg);
– Sunitinib drug administration calendar (2 weeks of treatment followed by one week of drug pause vs. 4 weeks of treatment followed by two weeks of drug pause);
– Pazopanib treatment cost.

**Figure 3:**
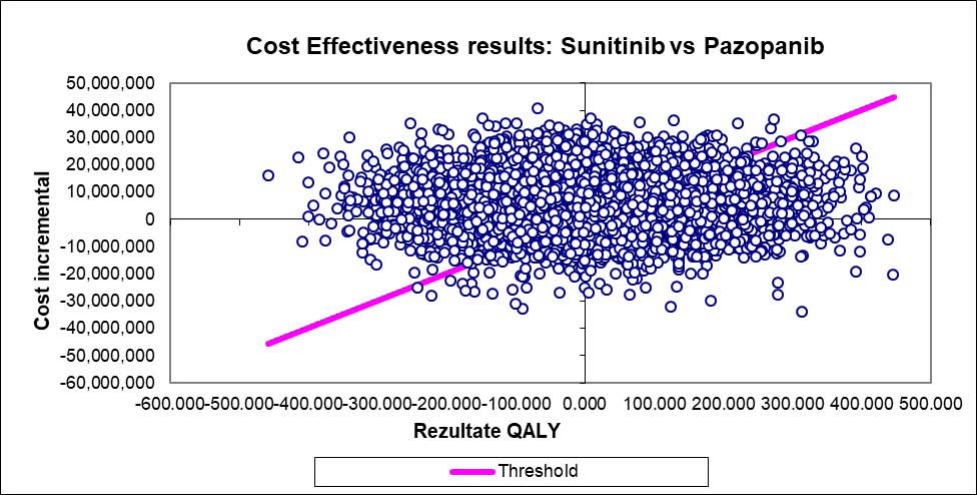
Distribution of intersection points for costs and utilities based on sensitivity analysis

The distribution in the cost-utility graph (Figure 3) shows a uniform dissipation of intersection points for the variables considered.

**Table 3:** Costs of therapeutic alternatives

Therapeutic alternatives	Treatment cost for 6 weeks (RON)
**Sunitinib**	
50 mg	19.748
37.5 mg	15.003
**Pazopanib**	
800 mg	15.507
600 mg	11.630
400 mg	7.753
**Second-line treatments**	
**Sorafenib**	9.482
**Everolimus**	20.090
**Bevacizumab (Includes treatment administration costs)**	29.897
**Temsirolimus**	19.552
**Axitinib**	25.105

**Table 4:** Medical services costs

Cost-type	Value (RON)
**Medical service**	
Hospitalization episode in the oncology department	998
Visiting the oncologist (ambulatory care)	20.4
Palliative service at home	65
Payment of intensive care services	5963
Payment for hospitalization episode with surgical intervention	2656
Palliative surgery	2305
**Laboratory tests**	
Hemogram	14
Set of medical analyses for metabolismevaluation	175.67
Oncologic markers	145
Thyroid function tests	40
**Imaging investigations**	
Thoracic or pelvic CT scan	175
Radiography	32
Echography	60
MRI	700
Bone scintigraphy	35

**Table 5:** Results of Sunitinib vs. Pazopanib

	Sunitinib	Pazopanib
Progression-Free Survival Life Years (PFSLY)	570.69	577.88
Overall Survival (Life Years)	721.32	733.04
Quality-Adjusted Life-Years (QALYs)	497.84	506.81
Costs of first-line treatment	66271079.45	57998473.94
Adverse events costs for first-line treatment	1127299.24	988869.31
Pre-progression costs	67398378.69	58987343.25
Costs of second-line treatment	12932711.77	14865966.38
Total costs without discount	80331090.46	73853309.63
Incremental results of Pazopanib vs Sunitinib		
ICER - PfLYs	—	(901318.06)
ICER - LYs	—	(553011.69)
ICER - QALYs	—	(722296.54)

**Table 6:** Cost-effectiveness and cost-utility ratio for the two therapeutic alternatives

	**Pazopanib vs. Sunitinib**
Incremental PFSLY	–7.19
Incremental LY	–11.71
Incremental QALYs	–8.97
Incremental cost	6477781
Incremental cost per PFSLY gained	–901.318
Incremental cost per LY gained	–553.012
Incremental cost per QALY gained	–722.297

## Discussions and Conclusions

This analysis provides information on the cost-effectiveness of 2 therapeutic alternatives reimbursed for the treatment of metastatic renal cancer in Romania as first-line of treatment alternatives. In line with the results of the COMPARZ study, administration of the 4-week treatment with a 2-week rest period and measurement of quality of life outcomes on the last day of the 4-week cycle may lead to alteration of the measurements. If the measurement of the quality of life was achieved during the therapeutic period, the results of the comparison of the two drugs might differ. Given that differences in clinical efficacy or quality of life for the two drugs are not very high, we estimate that the only major difference can be given by the differences between the compensation values for the two drugs studied. Also, the cost-effectiveness of the two evaluated alternatives has an impact on the subsequent costs for the following treatment lines as therapies reimbursed for second-line are considerably more expensive than the two alternatives studied.

## Conflict of Interest

The authors confirm that there are no conflicts of interest.
